# P-232. Prognostic Value of Routine CSF Markers in Cryptococcal Meningitis Among PLHIV: A Subanalysis from a Philippine Cohort

**DOI:** 10.1093/ofid/ofaf695.454

**Published:** 2026-01-11

**Authors:** Ma Jean Capulong Linsao, Rontgene Solante

**Affiliations:** San Lazaro Hospital, Manila, National Capital Region, Philippines; San Lazaro Hospital, Manila, National Capital Region, Philippines

## Abstract

**Background:**

Cryptococcal meningitis (CM) is a life-threatening opportunistic infection among people living with HIV (PLHIV), particularly in low- and middle-income countries. While diagnostic tools like India ink and cryptococcal antigen (CrAg) are widely used, the prognostic significance of routine cerebrospinal fluid (CSF) biomarkers remains unclear. This study evaluated associations between CSF parameters and in-hospital mortality in PLHIV with CM. This is a subanalysis of a previously presented five-year cohort on central nervous system infections in HIV from a national referral center in the Philippines.

**Figure:**
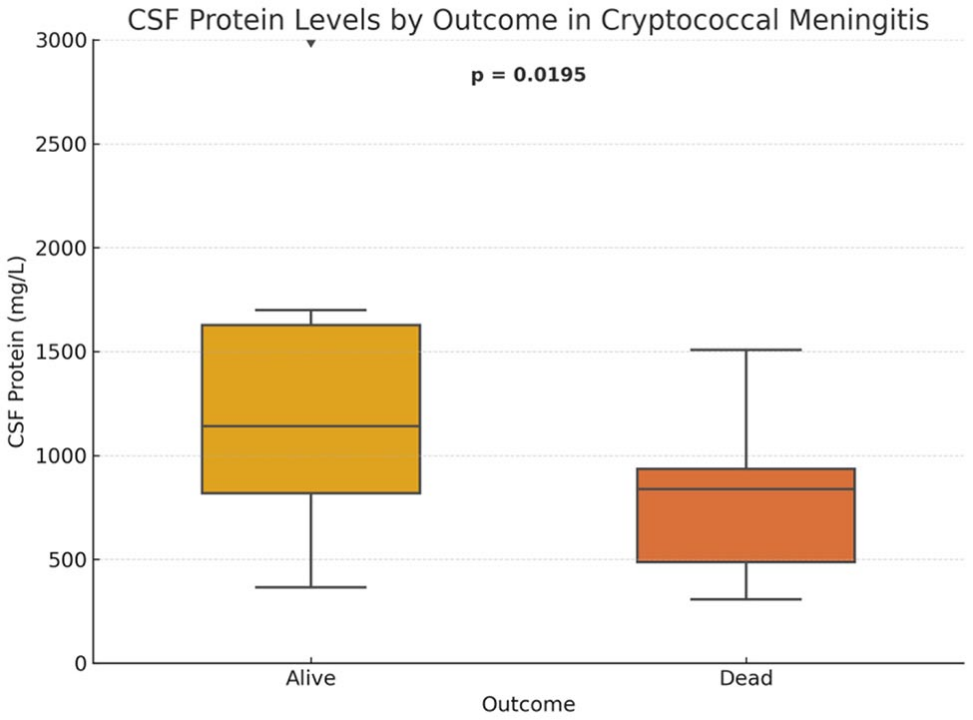
CSF Protein Levels by Outcome

Survivors had significantly higher CSF protein levels than non-survivors (p = 0.0195), suggesting preserved inflammatory response may be protective.

**Figure:**
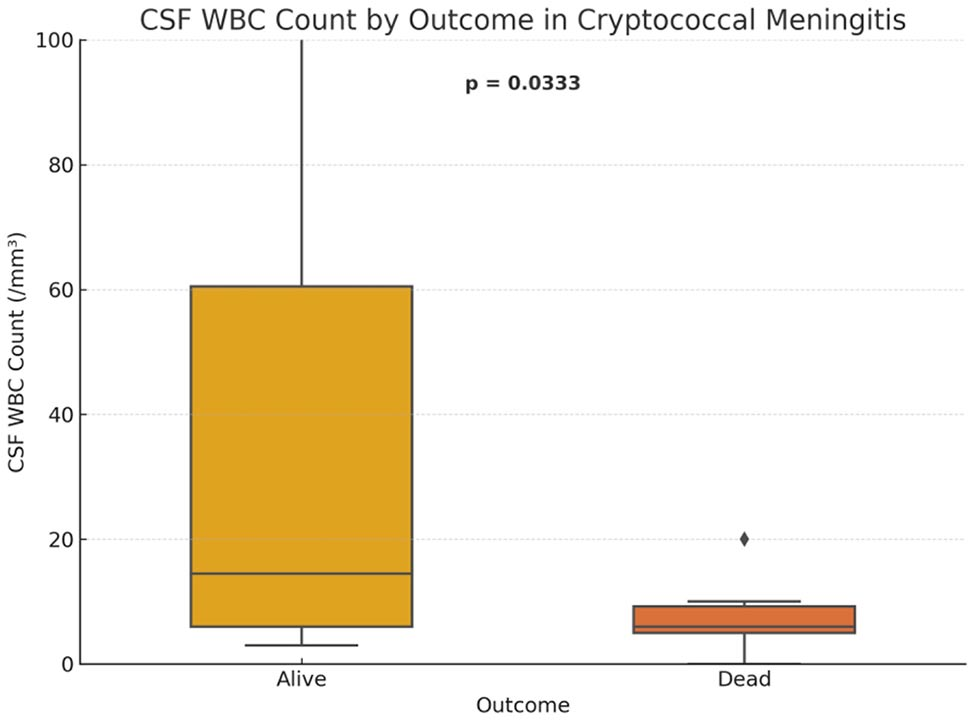
CSF WBC Count by Outcome

Median CSF white cell count was significantly higher among survivors (p = 0.0333), indicating stronger local immune activation.

**Methods:**

We retrospectively reviewed adult PLHIV admitted with CM at San Lazaro Hospital, Manila, from 2018 to 2022. Demographics, CSF parameters (glucose, protein, white blood cell [WBC] count), CD4 count, India ink, and CrAg results were collected. In-hospital mortality was the primary outcome. Statistical analysis included Mann-Whitney U, Fisher’s exact test, and receiver operating characteristic (ROC) analysis.

**Figure:**
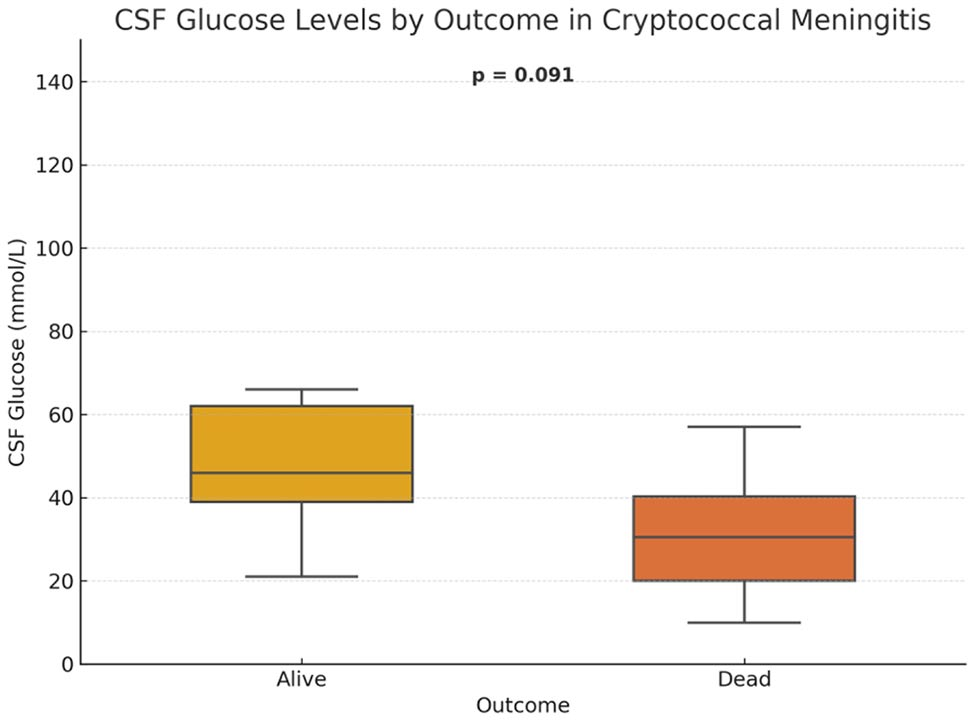
CSF Glucose Levels by Outcome

No statistically significant difference in CSF glucose was observed between survivors and non-survivors (p = 0.091), though a downward trend was seen in deaths.

**Figure:**
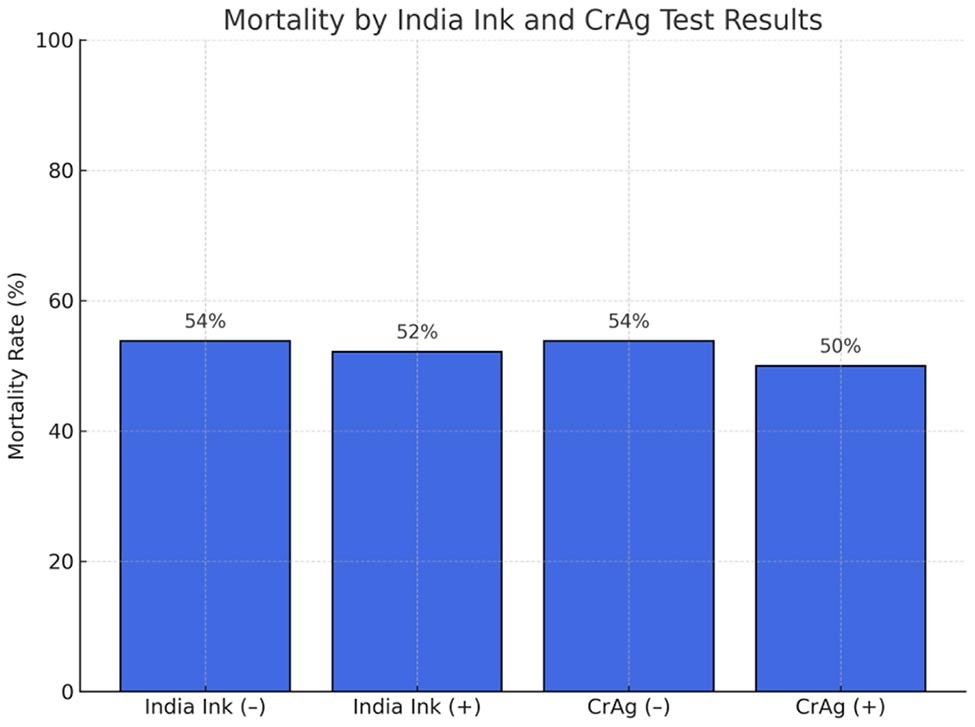
Mortality by India Ink and CrAg Results

Mortality rates did not differ significantly by India Ink or CrAg positivity (both p = 1.0), though clinical utility for diagnosis remains high.

**Results:**

Thirty-six patients were included (mean age: 30.5 years); 19 (53%) died. ROC analysis showed poor discriminatory value for CSF glucose (area under the curve [AUC] 0.26), protein (AUC 0.24), WBC (AUC 0.27), and CD4 count (AUC 0.32). India ink and CrAg positivity were not significantly associated with mortality (p=1.0). Survivors had significantly higher CSF protein (median 1114 vs 851 mg/L; p=0.0195) and WBC counts (median 14 vs 6 /mm³; p=0.0333).

**Conclusion:**

In HIV-associated CM, higher CSF protein and WBC counts were associated with survival, suggesting that preserved inflammatory response may influence outcomes. Basic CSF markers could aid early prognostication and risk stratification in resource-limited settings. These findings may be applicable to similar high-burden, low-resource contexts where advanced diagnostics are limited.

**Disclosures:**

All Authors: No reported disclosures

